# Further New Highly Oxidative Cembranoids from the Hainan Soft Coral *Sarcophyton trocheliophorum*

**DOI:** 10.1007/s13659-016-0088-4

**Published:** 2016-03-15

**Authors:** Wen-Ting Chen, Lin-Fu Liang, Xu-Wen Li, Wei Xiao, Yue-Wei Guo

**Affiliations:** State Key Laboratory of Drug Research, Shanghai Institute of Materia Medica, Chinese Academy of Sciences, 555 Zu Chong Zhi Road, Zhang Jiang High-Tech Park, Shanghai, 201203 People’s Republic of China; College of Material Science and Engineering, Central South University of Forestry and Technology, 498 South Shaoshan Road, Changsha, 410004 People’s Republic of China; Jiangsu Kanion Pharmaceutical Co., Ltd., Lianyungang, 222001 People’s Republic of China

**Keywords:** Soft coral, *Sarcophyton trocheliophorum*, Cembranoids, Sarcophytrol, Structure elucidation

## Abstract

**Abstract:**

Three new highly oxidative cembranoids, sarcophytrols D–F (**1**–**3**), were obtained from the South China Sea soft coral *Sarcophyton trocheliophorum*, along with two known related ones (**4** and **5**). Their structures were elucidated by extensive spectroscopic analyses and by comparison with literature data. The discovery of these new secondary metabolites enriched the family of cembranoids deduced from the title animal.

**Graphical Abstract:**

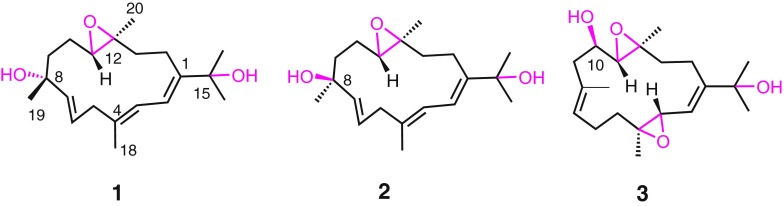

## Introduction

Soft corals (phylum Cnidaria, class Anthozoa, subclass Octocorallia, order Alcyonacea) often equal or exceed the total coverage of scleractinian corals in coral reef ecosystems [1–4]. The family Alcyoniidae within the order Alcyonacea contains the productive genus *Sarcophyton* [5, 6]. Till now, nearly 16 species of soft corals of the genus *Sarcophyton*, from various geographical areas, have been chemically investigated, being reported to comprise diverse diterpenes with different skeletons (cembrane, sarsolenane, capnosane, etc.) [5–9], biscembranoids [10–12], polyhydroxylated sterols [13, 14], and other related metabolites [15, 16].

*Sarcophyton* species are prolific in the South China Sea. In the course of our search for bioactive substances from Chinese marine organisms, *Sarcophyton trocheliophorum* of the coast of Yalong Bay, Hainan Province, has been collected and chemically investigated, which were found to encompass numerous cembranoids with a variety of oxidation and cyclization patterns, including two unprecedented structures, methyl sarcotroates A and B [7–9, 12, 17]. In addition, many of them exhibited significant inhibitory activities against human PTP1B enzyme [7, 8, 17]. Recently, in order to find more chemically appealing and biologically active cembrane-based metabolites, *S. trocheliophorum* was re-collected from the same location while in a different growing period, and a further chemical investigation yielded three new highly oxidative compounds named sarcophytrols D–F (**1**–**3**), along with two known ones (**4** and **5**). Details of the isolation, structure elucidation and biological study of these secondary metabolites are reported herein.

## Results and discussion

Samples of *S. trocheliophorum* (400 g, dry weight) were extracted exhaustively with acetone, and the extract was partitioned between H_2_O and Et_2_O. The Et_2_O soluble fraction was subjected to silica gel chromatography (light petroleum ether/acetone gradient). The lower polar fractions were subsequently purified on repeated column chromatography (silica gel, Sephadex LH-20, reversed phase-C_18_ silica gel and semi preparative-HPLC) to afford five pure metabolites, compounds **1**–**5**. A preliminary NMR analysis revealed that all the new molecules shared the same cembrane skeleton. Among them, two known compounds were readily identified as 11,12-epoxy-1(*E*),3(*E*),7(*E)*-cembratrien-15-ol (**4**) [18] and sinugibberol (**5**) [19] by comparison of their spectral data and [*α*]_D_ values with those reported in the literature.

Sarcophytrol D (**1**) was obtained as colorless oil. The molecular formula was established as C_20_H_30_O_3_ by HRESIMS (*m/z* 343.2248 [M + Na]^+^), sixteen mass units more than that of compound **4** [18]. In fact, their NMR spectra were similar, except for signals appeared in the downfield area. Differed from **4**, the ^1^H and ^13^C NMR (Tables 1 and 2) data of **1** showed the presence of a *trans*-disubstituted double bond (*δ*_H_ 5.75, d, *J* = 15.7 Hz; *δ*_H_ 5.68, ddd, *J* = 15.7, 7.4, 5.4 Hz). In its ^1^H–^1^H COSY, the cross-peaks between H-5 (*δ*_H_ 2.83, 2.75) and H-6 (*δ*_H_ 5.68), between H-6 and H-7 (*δ*_H_ 5.75) indicated the linkage of C-5 to the *trans*-double bond. Furthermore, HMBC correlations from Me-19 (*δ*_H_ 1.40) to C-7 (*δ*_C_ 140.4) and from Me-18 (*δ*_H_ 1.78) to C-5 (*δ*_C_ 41.47) confirmed the position of the double bond. In addition, Me-19 of compound **1** has been upfield shifted (from *δ*_H_ 1.67 in **4** to 1.40 in **1**). Based on these NMR and MS variations, the hydroxylation at C-8 accompanying the double-bond migration from Δ^7(8)^ to Δ^6(7)^ in the structure of **1** were supported. Assignments of ^1^H and ^13^C NMR signals of **1** were made by the application of detailed 2D NMR analysis.

**Table 1:** ^1^H NMR data [*δ*_H_ (mult., *J* in Hz)] for compounds **1**–**3** ^a^

No.	1	2	3
2	6.30, d, (11.4)	6.32, d (11.3)	5.34, d (6.4)
3	5.86, d, (11.4)	5.73, d (11.3)	3.36, d (6.4)
5	2.83, dd (17.7, 7.4) 2.75, dd (17.7, 5.4)	2.84, dd (17.7, 9.2) 2.78, dd (17.7, 4.6)	2.07, m 1.61, m
6	5.68, ddd (15.7, 7.4, 5.4)	5.81, ddd (15.6, 9.2, 4.6)	2.22, m 2.02, m
7	5.75, d (15.7)	5.71, dd (15.6, 1.5)	5.26, t (5.8)
9	2.00, ddd (12.7, 8.3, 1.9) 1.70, ddd (12.7, 11.5, 9.1)	1.96, m 1.79, m	2.44, dd (6.3, 13.7) 2.30, dd (3.4, 13.7)
10	1.92, m 1.63, m	2.03, m 1.52, m	3.70, ddd (8.7, 7.7, 4.0)
11	3.08, t (6.2)	3.22, dd (7.4, 4.3)	2.90, d (8.7)
13	1.94, m 1.10, dd (12.9, 6.6)	2.22, m 0.79, m	2.11, m 1.44, dd (2.3, 12.5)
14	2.33, ddd (13.2, 11.1, 6.8) 2.22, dd (11.1, 3.0)	2.22, m	2.21, m 2.16, m
16	1.36, s	1.36, s	1.36, s
17	1.36, s	1.36, s	1.36, s
18	1.78, s	1.78, s	1.25, s
19	1.40, s	1.36, s	1.76, s
20	1.25, s	1.24, s	1.30, s

^a^Bruker-DRX-500 spectrometer (500 MHz) in CDCl_3_; chemical shifts (ppm) referred to CHCl_3_ (*δ*_H_ 7.26); assignments were deduced from analysis of 1D and 2D NMR spectra

**Table 2:** ^13^C NMR data (*δ*_C_, mult.) for compounds **1**–**3** ^a^ and **5** ^b^

No.	1	2	3	5
1	147.6, s	147.3, s	150.9, s	150.9, s
2	117.4, d	117.6, d	121.0, d	125.8, d
3	119.8, d	119.6, d	58.7, d	59.3, d
4	138.2, s	138.4, s	61.9, s	61.7, s
5	41.4, t	41.4, t	37.3, t	36.8, t
6	125.0, d	123.6, d	22.1, t	22.3, t
7	140.4, d	141.0, d	128.6, d	121.2, d
8	72.6, s	72.8, s	133.1, s	134.9, s
9	40.1, t	38.9, t	43.8, t	40.2, t
10	24.0, t	23.8, t	69.1, d	26.0, d
11	64.4, d	63.6, d	65.1, d	62.1, d
12	62.4, s	61.4, s	62.4, s	61.2, s
13	41.5, t	42.0, t	39.2, t	38.0, t
14	24.6, t	25.3, t	24.7, t	24.5, t
15	73.8, s	73.8, s	73.4, s	73.4, s
16	29.2, q	29.3, q	29.7, q	29.7, q
17	29.2, q	29.7, q	29.7, q	29.7, q
18	18.5, q	18.5, q	18.5, q	18.0, q
19	28.8, q	31.3, q	17.9, q	14.7, q
20	15.8, q	15.7, q	18.0, q	16.1, q

^a^Bruker-DRX-500 spectrometer (125 MHz) in CDCl_3_; chemical shifts (ppm) referred to CHCl_3_ (*δ*_C_ 77.0); assignments were deduced from analysis of 1D and 2D spectra

^b^Data reported in the literature [19]

**Fig. 1:**
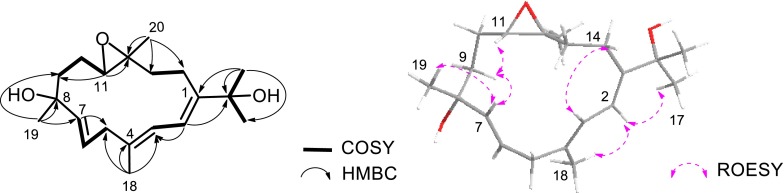
Key COSY, HMBC and ROESY correlations for compound **1**

**Fig. 2:**
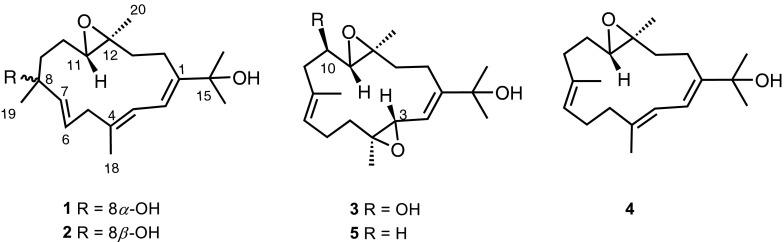
Chemical structures of compounds **1**–**5**

As for the relative configuration of compound **1**, according to the ROESY correlations between H-2 (*δ*_H_ 6.30)/Me-17 (*δ*_H_ 1.36), H-2/Me-18 (*δ*_H_ 1.78) and H-3 (*δ*_H_ 5.86)/H-14b (*δ*_H_ 2.22) (Fig. 1), the olefinic geometries were assigned to 1*E* and 3*E*. The ^13^C NMR chemical shift of Me-20 (*δ*_C_ < 20 ppm) and the absence of the ROESY correlations of H-11 (*δ*_H_ 3.08)/Me-20 (*δ*_H_ 1.25) indicated the *trans*-configuration of the epoxy group at C-11/C-12 in **1**, which was the same as that in **4**. Additional ROESY interactions of H-11/H-7, H-7/H-9b (*δ*_H_ 1.70), H-9b/H-11 suggested these three protons were co-facial, assigned tentatively as *β*-orientation. Thus, Me-19 was accordingly *β*-oriented due to the diagnostic cross-peak of Me-19 (*δ*_H_ 1.40) with H-7, allowing the determination of the structure of **1** as showed in Fig. 2, which was the Δ^6(7)^-8*α*-hydroxyl derivative of **4**.

**Fig. 3:**
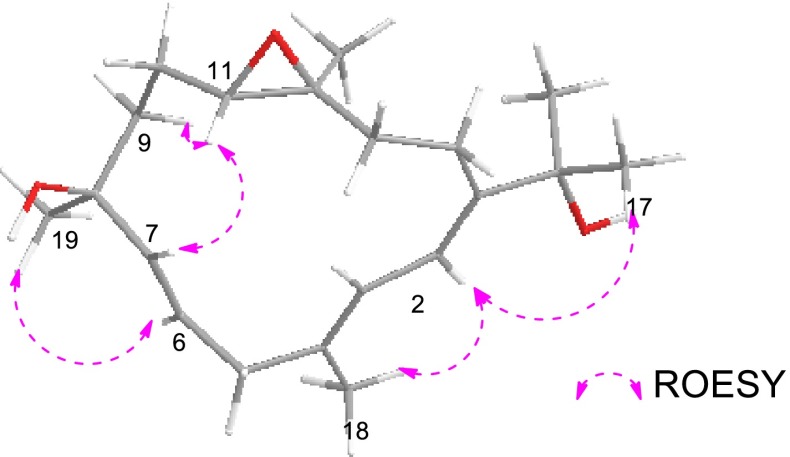
Key ROESY correlations for compound **2**

Sarcophytrol E (**2**) was also obtained as colorless oil. The molecular formula, C_20_H_30_O_3_, established by HRESIMS (*m/z* 343.2243 [M + Na]^+^), was identical to that of **1**. A detailed 2D NMR analysis of **2** and careful comparison with the NMR data of **1** (Tables 1 and 2) revealed that their structures were almost the same. In fact, the only differences between **2** and **1** were the C-19 signal downfield shifted in the ^13^C NMR spetrum (*δ*_C_ 28.8 in **1** and *δ*_C_ 31.3 in **2**), while the C-6 and C-9 signals upfield shifted (*δ*_C_ 125.0, 40.1 in **1** and *δ*_C_ 123.6, 38.9 in **2**, respectively). The observed differences can be rationalized when the two compounds are C-8 epimers, which was confirmed by the ROESY interaction of H-6 (*δ*_H_ 5.81)/Me-19 (*δ*_H_ 1.36) (Fig. 3). Since the hydroxyl group at C-8 of **1** was *α*-oriented, the opposite configuration at this center is therefore tentatively suggested for **2**.

**Fig. 4:**
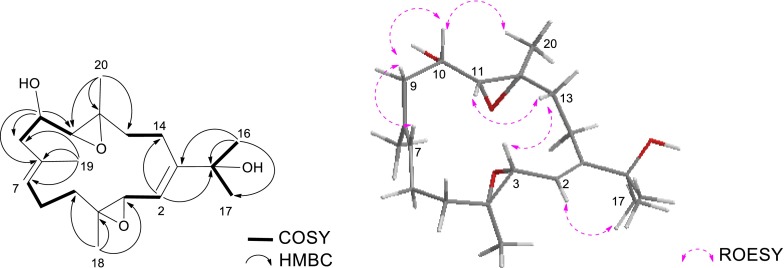
Key COSY, HMBC and ROESY correlations for compound **3**

The HRESIMS of sarcophytrol F (**3**) established the molecular formula C_20_H_32_O_4_ (*m/z* 359.2188 [M + Na]^+^), 16 mass units more than that of sinugibberol (**5**) [19]. The ^1^H and ^13^C NMR data of **3** showed great similarity as those of **5** (Tables 1 and 2), some minor differences were observed in relation to the functional group. The presence of a secondary hydroxyl group in the molecule was readily recognized by a signal resonating at *δ*_H_ 3.70 (1H, ddd, *J* = 8.7, 7.7, 4.0 Hz) in its ^1^H NMR spectrum, and by a carbon signal at *δ*_C_ 69.1 (CH) in the ^13^C NMR and DEPT spectra. The oxygenated methine proton was secured at C-10 by a COSY cross-peak between H-9 (*δ*_H_ 2.44, 2.30) and H-11 (*δ*_H_ 3.57), and the HMBC correlations with C-8, C-9 and C-11 (Fig. 4). Due to the presence of the 10-OH, ^13^C NMR chemical shifts of C-9 to C-12 were all reasonably downfield shifted with respect to those of **5**.

Finally, the relative configuration of **3** was determined by ROESY experiment and by comparison of the NMR data with **5** (Fig. 4). The ^13^C NMR chemical shift of Me-18 and Me-20 (*δ*_C_ < 20 ppm) and the absence of ROESY correlations of Me-18 (*δ*_H_ 1.25)/H-3 (*δ*_H_ 3.36), and Me-20 (*δ*_H_ 1.30)/H-11 (*δ*_H_ 2.90), indicated the *trans*-configurations of these two epoxy groups at C-3/C-4 and C-11/C-12 in **3**, which were the same as those in **5**. The similar ^13^C NMR data of C-3 and C-4 in **3** and **5** further confirmed the same stereochemistry of this epoxy group. Furthermore, the ROESY correlations of H-11/H-13a (*δ*_H_ 1.44), H-3/H-13a, suggested that H-11 and H-3 were co-facial, leading to the determination of the relative configurations at C-11 and C-12 where the other epoxy group resided on. In addition, the ROESY correlations of H-10/Me-20 indicated that 10-OH and H-11 were co-facial. Due to the scarcity of material, the modified Mosher’s method could not be able to apply for the determination of the absolute configuration in C-10 position in **3** at this moment. Thus the structure of sarcophytrol F (**3**) was tentatively determined as 10-hydroxyl derivative of **5**.

All the compounds were tested for the cytotoxic activities and inhibitory activities against human protein tyrosine phosphatase 1B (PTP1B), a key target for the treatment of type-II diabetes and obesity [20]. Unfortunately, none of them showed inhibitory effects toward the above bioassays. Further study should be conducted to understand the real biological/ecological role of these metabolites in the life cycle of the animal, as well as to carry out other biological evaluations such as antioxidant, anti-inflammatory, anti-fouling activities, etc.

## Experimental Section

### General Experimental Procedures

Optical rotations were measured on a Perkin-Elmer 341 polarimeter. HRESIMS spectra were recorded on a Waters-Micromass Q-TOF Ultima Global electrospray mass spectrometer. NMR spectra were measured on a Bruker-DRX-500 spectrometer with the residual CHCl_3_ (*δ*_H_ 7.26 ppm, *δ*_C_ 77.0 ppm) as internal standard. Chemical shifts are expressed in *δ* (ppm) and coupling constants (*J*) in Hz. ^1^H and ^13^C NMR assignments were supported by ^1^H-^1^H COSY, HSQC, and HMBC experiments. Commercial silica gel (Qing Dao Hai Yang Chemical Group Co., 200–300 and 400–600 mesh), C_18_ reversed-phase silica gel (150–200 mesh, Merck) and Sephadex LH-20 (Amersham Biosciences) were used for column chromatography. Semi-preparative HPLC (Agilent 1100 series liquid chromatography using a VWDG1314A detector at 210 nm and a semi-preparative ODS-HG-5 [5 μm, 10 mm (i.d.) × 25 cm] column was also employed. Pre-coated silica gel GF_254_ plates (Qing Dao Hai Yang Chemical Group Co. Ltd. Qingdao, People’s Republic of China) were used for analytical thin-layer chromatography (TLC). All solvents used were of analytical grade (Shanghai Chemical Reagents Company, Ltd.).

### Animal Material

The soft corals *S. trocheliophorum* were collected by scuba at Yalong Bay, Hainan Province, China, in February 26, 2006, at a depth of −15 to −20 m, and identified by Professor R.-L. Zhou of South China Sea Institute of Oceanology, Chinese Academy of Sciences. The voucher sample is deposited at the Shanghai Institute of Materia Medica, CAS, under registration No. YAL-4.

### Extraction and Isolation

The lyophilized bodies of *S. trocheliophorum* (400 g, dry weight) were minced into pieces and exhaustively extracted with Me_2_CO at room temperature (3 × 1 L). The solvent-free Me_2_CO extract was partitioned between Et_2_O and H_2_O. The organic phase was evaporated under reduced pressure to give a dark brown residue (10 g), which was subjected to silica gel column chromatography (CC) and eluted with petroleum ether (PE) in Me_2_CO (0–100 %, gradient) to yield 14 fractions (A–M).

Fraction D was chromatographed over silica gel (PE/Et_2_O, 1:1) to give pure **4** (32.1 mg). Fraction F was subjected to Sephadex LH-20 CC (PE/CH_2_Cl_2_/MeOH, 2:1:1), followed by repeated silica gel CC (PE/CH_2_Cl_2_, 20:1) to yield 6 sub-fractions (F1-F6). F2 gave compound **5** (2.3 mg) after silica gel CC (PE/CH_2_Cl_2_, 10:1). Fraction G was chromatographed over Sephadex LH-20 (CHCl_3_/MeOH, 1:1), followed by ODS (MeOH/H_2_O, 50:50–90:10) to yield 10 sub-fractions (G1-G10). After purification by RP-HPLC (MeOH/H_2_O, 88:12, 2.0 mL/min), G5 yielded pure **1** (2.4 mg, *t*_R_ 6.3 min). Fraction H gave compound **3** (2.3 mg, *t*_R_ 12.5 min) after Sephadex LH-20 CC (PE/CHCl_3_/MeOH, 2:1:1), CC on ODS (MeOH/H_2_O, 45:55–90:10) and RP-HPLC (MeOH/H_2_O, 73:27, 2.0 mL/min). Fraction I was subjected to Sephadex LH-20 CC (PE/CHCl_3_/MeOH, 2:1:1) to give five sub-fractions (I1-I5). Fraction I4 was first split by CC on ODS (MeOH/H_2_O, 50:50–90:10) and then purified by silica gel (CHCl_3_/MeOH, 50:1) to afford pure **2** (3.1 mg).

Sarcophytrol D (**1**): colorless oil; [*α*] +18.6 (*c* 0.07, MeOH); ^1^H and ^13^C NMR data, see Tables 1 and 2; HRESIMS *m*/*z* 343.2248 [M + Na]^+^ (calcd for C_20_H_32_O_3_Na, 343.2249).

Sarcophytrol E (**2**): colorless oil; [*α*] +48.6 (*c* 0.10, MeOH); ^1^H and ^13^C NMR data, see Tables 1 and 2; HRESIMS *m*/*z* 343.2243 [M + Na]^+^ (calcd for C_20_H_32_O_3_Na, 343.2249).

Sarcophytrol F (**3**): colorless oil; [*α*] −12.7 (*c* 0.20, MeOH); ^1^H and ^13^C NMR data, see Tables 1 and 2; HRESIMS *m/z* 359.2188 [M + Na]^+^ (calcd for C_20_H_32_O_4_Na, 359.2188).
